# Underestimating the frequency, strength and cost of antipredator responses with data from GPS collars: an example with wolves and elk

**DOI:** 10.1002/ece3.896

**Published:** 2013-11-26

**Authors:** Scott Creel, John A Winnie, David Christianson

**Affiliations:** 1 Department of Ecology, Montana State University310 Lewis Hall, Bozeman, Montana, 59717, U.S.A; 2 School of Natural Resources and the Environment, University of Arizona325 Biological Sciences East, Tucson, Arizona, 85721, U.S.A

**Keywords:** Antipredator behavior, detection bias, elk, GPS, nonconsumptive effects, risk effects, wolf

## Abstract

Field studies that rely on fixes from GPS-collared predators to identify encounters with prey will often underestimate the frequency and strength of antipredator responses. These underestimation biases have several mechanistic causes. (1) *Step bias*: The distance between successive GPS fixes can be large, and encounters that occur during these intervals go undetected. This bias will generally be strongest for cursorial hunters that can rapidly cover large distances (e.g., wolves and African wild dogs) and when the interval between GPS fixes is long relative to the duration of a hunt. Step bias is amplified as the path travelled between successive GPS fixes deviates from a straight line. (2) *Scatter bias*: Only a small fraction of the predators in a population typically carry GPS collars, and prey encounters with uncollared predators go undetected unless a collared group-mate is present. This bias will generally be stronger for fission–fusion hunters (e.g., spotted hyenas, wolves, and lions) than for highly cohesive hunters (e.g., African wild dogs), particularly when their group sizes are large. Step bias and scatter bias both cause underestimation of the frequency of antipredator responses. (3) *Strength bias*: Observations of prey in the absence of GPS fix from a collared predator will generally include a mixture of cases in which predators were truly absent and cases in which predators were present but not detected, which causes underestimation of the strength of antipredator responses. We quantified these biases with data from wolves and African wild dogs and found that fixes from GPS collars at 3-h intervals underestimated the frequency and strength of antipredator responses by a factor >10. We reexamined the results of a recent study of the nonconsumptive effects of wolves on elk in light of these results and confirmed that predation risk has strong effects on elk dynamics by reducing the pregnancy rate.

## Introduction

Predators affect the demography and population dynamics of prey by direct killing and by altering prey behavior. Virtually all prey species alter their behavior in response to predation risk, for example by increasing vigilance (Brown and Kotler 2004), altering group size (Creel and Winnie 2005), or retreating to safe habitats (Sih 1997). In many cases, antipredator responses are known to carry costs by decreasing feeding rates (Kotler et al. 1991; Brown and Kotler 2004), causing changes in diet (Christianson and Creel 2010), or provoking physiological stress responses (Clinchy et al. 2004, 2013). Experimental studies have repeatedly shown that predation risk can reduce prey growth and reproduction (Werner et al. 1983; Peckarsky et al. 1993; Relyea and Werner 1999; Zanette et al. 2011) and that risk effects can comprise a substantial fraction of the total effect of predators on prey dynamics (Preisser et al. 2005). While these experiments clearly show that risk effects can be strong, field studies with natural variation in predation risk are needed to measure their strength in the wild, and to learn more about the variables that affect the balance between direct predation and risk effects (Heithaus and Dill 2006; Schmitz 2008; Creel 2011). To date, very few field studies have measured the demographic costs of antipredator responses to natural variation in predation (Creel and Christianson 2008). This situation arises at least in part because behavioral responses vary at the individual level on short time scales, while demographic responses are measured at the population level over longer time scales. These issues of scale make it difficult to detect a chain of relationships from predator presence to behavioral responses to physiological and demographic consequences. Moreover, predation risk is one of many factors that may simultaneously affect prey demography, which complicates inferences about causation.

In the Northern Rocky Mountains, wolves (*Canis lupus*) are generally the dominant predator of elk (*Cervus canadensis*), and elk are the dominant prey of wolves in systems where elk are common (Hebblewhite et al. 2002; Smith et al. 2004; Winnie and Creel 2007; Griffin et al. 2011). The translocation of wolves to parts of the Northern Rocky Mountains in the mid-1990s provided an unusual opportunity to pseudoexperimentally test the effects of predation risk on elk behavior, ecology, physiology, demography, and dynamics, either by comparison of data from before and after local wolf colonization (e.g., see Figs. 6 and 7 below) or by comparison of populations that were colonized by wolves to populations that were not (e.g., See Figs. 6 and 7 below). Studies have also taken advantage of variation between populations in wolf and elk density to test for antipredator responses without dichotomizing predation risk into the categories “present” and “absent” (e.g., Creel et al. 2007; Liley and Creel 2008). A series of studies using these approaches has found that, like most animals (Caro 2005), elk use a broad set of antipredator responses to reduce the risk of direct predation. In the presence of wolves, elk alter patterns of aggregation (Creel and Winnie 2005), vigilance (Winnie and Creel 2007; Creel et al. 2008), foraging behavior (Winnie and Creel 2007), habitat selection (Creel et al. 2005), and diet selection (Christianson and Creel 2008, 2010). Parallel to these antipredator responses, elk nutritional condition has been found to decline, including a decrease in energy intake equivalent to 27% of maintenance requirements and an increase in endogenous protein catabolism (Christianson and Creel 2010). Data from 10 elk populations show that pregnancy rates have decreased by 24% to 43% following wolf recolonization, while reproduction has generally remained unchanged in nearby populations that were not colonized by wolves (Zager et al. 2005; Creel et al. 2007, 2011; Garrott et al. 2009; Stephenson 2010; White et al. 2011). Decreased pregnancy rates have been detected with large sample sizes (e.g., *N* = 1489 in Creel et al. 2007) using a broad range of methods that include fecal progesterone assays, serum progesterone assays, and serum PSPB assays, and the correlation between results from different methods is very strong (Creel et al. 2007, 2011). Decreased pregnancy rates correlate strongly with reduced calf recruitment and altered population dynamics in these populations (e.g., see Figs. 6 and 7 below and Creel et al. 2007, 2011). This extensive body of knowledge drawn from 18 years of data relating the responses of elk to the presence of wolves suggests that natural variation in predation risk has strong effects on the behavior, ecology, physiology, demography, and dynamics of elk, as has been shown experimentally with other taxa (Peckarsky et al. 1993; Preisser et al. 2005; Zanette et al. 2011).

Recently, Middleton et al. (2013a) estimated the frequency and strength of antipredator responses in two adjacent Wyoming elk herds with different levels of predation risk from wolves. They quantified the frequency of encounters with wolves (and the strength of behavioral responses to these encounters) by relating data from elk to GPS fixes collected at 3-h intervals from a subset (∼30%) of the wolves on their study site that carried GPS radiocollars. Although these methods showed that “when wolves approached within 1 km, elk increased their rates of movement, displacement, and vigilance”, Middleton et al. (2013a) concluded that the frequency and strength of responses to wolves were too small to affect elk demography or dynamics. However, this type of data from GPS collars can underestimate the frequency and strength of antipredator responses (and thus underestimate risk effects) for three reasons. First, encounters with prey go undetected if they occur in the intervals between GPS fixes. Second, encounters between prey and uncollared predators go undetected, unless the predator is with a collared group-mate. Both of these effects will generally cause underestimation of the rate of encounter between predators and prey. Third, for methods that rely solely on fixes from GPS collars to describe risk, observations of prey under “safe” conditions will generally include a mixture of cases in which predators were truly absent and cases in which predators were present but not detected (see discussion in Creel and Winnie 2005; Winnie and Creel 2007). Unless the spatiotemporal coverage of GPS fixes is comprehensive, data from prey under safe conditions will be contaminated to some degree by data with undetected risk. Undetected risk will cause underestimation of the strength of responses when predators are present, in a manner that is logically similar to well-understood detection problems that motivated the development of occupancy models and mark-recapture analysis. In the context of occupancy models, failure to adjust for nondetection causes underestimation of a species' occurrence (MacKenzie 2006). In the context of mark-recapture analysis, failure to account for nondetection causes underestimation of survival rate or population size (Nichols 1992). Following the same logic, it is clear that failure to account for undetected predator encounters will cause underestimation of the strength of antipredator responses.

Empirical analysis is necessary to determine the magnitude of these biases. Here, we first identify and explain three general mechanisms that cause underestimation bias. We then use data from wolves and African wild dogs to estimate their magnitude. In light of the results, we critically reevaluate Middleton et al. (2013a) inference that responses of elk to wolves are too infrequent and weak to provoke demographic costs.

## “Step bias” in estimates of encounter frequency

**Figure 1:**
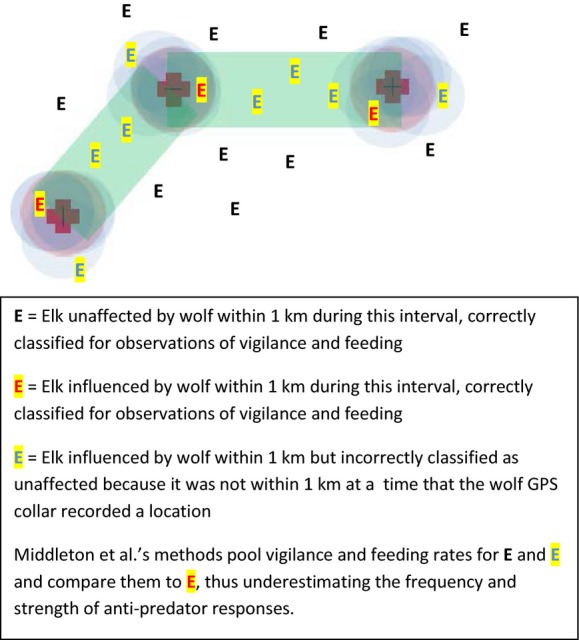
Using uncorrected data from GPS collared wolves, Middleton et al. (2013a) recorded a predator–prey encounter for elk within a defined radius (red circles) of a GPS-collared wolf at the instant that its collar recorded a location (red crosses). Here, green bands denote the continued existence of collared wolves between GPS locations. Middleton also made no adjustment for independent movements by uncollared wolves, which constituted approximately 70% of the population (the exact proportion was not reported). Blue circles show a realistic distribution of pack mates at the time of a GPS location (see Fig. 4 for details). The method employed by Middleton et al. (2013a) (red) underestimates the actual area of encounter (red, green, and blue) and consequently underestimates the frequency of encounter. Elk observed in the blue and green areas would be incorrectly categorized as “not encountering” wolves, leading to underestimation of the magnitude of responses to wolf encounters (Winnie and Creel 2007). Finally, Middleton aggregated data on elk behavior for 24 h following an encounter, underestimating the strength of any response lasting <24 h.

GPS radiocollars are larger and heavier than conventional VHF collars, and the GPS battery has a strong effect on size and weight. To optimize the trade-off between the bulk of a GPS collar and its duration of service, it is common to increase the interval between fixes, allowing the collar to remain in low-amperage mode for longer periods. For example, Middleton et al. (2013a) programmed wolf GPS collars to attempt a fix once every 3 h. During an interval of several hours between fixes, the distance travelled by a large carnivore can be substantial, and encounters that occur during these movements go undetected (Fig. 1). We term the failure to detect encounters that occur between fixes “step bias”, because its magnitude is determined by the length of the steps between fixes, relative to the radius over which a predator affects prey behavior. This bias will be strong for predators that can move large distances during hunting periods (e.g., wolves and African wild dogs), when the interval between GPS fixes is long relative to the duration of a hunt, and for predators that cause antipredator responses only when they are close to prey (and hence for prey that has short flight distances).

## “Scatter bias” in estimates of encounter frequency

Because GPS radiocollars are substantially more expensive than conventional VHF collars, only a small fraction of the predators in a population typically carry GPS collars, so prey encounters with uncollared predators go undetected unless they are with a collared group-mate (Fig. 1). We term the failure to detect encounters with uncollared predators “scatter bias”, because its magnitude is determined by the distance between collared and uncollared predators, relative to the radius over which a predator affects prey behavior. This bias will be strongest for solitary hunters (e.g., striped hyenas: Wagner et al. 2008), but can also be strong for fission–fusion hunters in which subsets of a social group hunt independently of other group mates (e.g., spotted hyenas, wolves and lions), particularly when their group sizes are large. Scatter bias will be weaker for highly cohesive hunters such as African wild dogs.

## Bias in estimated strength of behavioral responses to predator presence

If the frequency of predator–prey encounters is underestimated, then the strength of antipredator responses will also be underestimated. For example, Middleton et al. (2013a) measured the strength of antipredator responses by comparing the behavior of elk that were within a defined radius of a wolf GPS fix to the behavior of elk that were not within that radius. There is little ambiguity about the sample of elk that were known to be near a GPS fix, but the sample of prey under “safe” conditions is a mixture of cases in which predators were truly absent and cases in which predators were present but not detected (Fig. 1). If a study relies solely on GPS telemetry to detect encounters, then one must consider the possibility that the sample of observations of “safe” prey is contaminated to some degree by observations conducted when a predator was present but no GPS collar recorded a location. This contamination causes underestimation of the strength of behavioral responses. If one detects strong antipredator responses to risk in such data, the result is conservative in the sense of underestimating the true magnitude of the response (see discussion in Creel et al. 2005; Winnie and Creel 2007).

Here, we used data on the movements of wolves and African wild dogs (*Lycaon pictus*) to quantify these biases for cursorial, pack-hunting canids. In light of these results, we reevaluated Middleton et al. (2013a) inference that the presence of wolves has limited effects on elk movements, behavior, and demography.

## Methods

### Step bias

We measured step bias using data on the movements of African wild dogs in the Selous Game Reserve, Tanzania (Creel and Creel 2002), and Liuwa National Park, Zambia. Using data from wild dogs to quantify step bias makes our analysis less specific to wolves, but more generally applicable to cursorial canids. It should be noted that differences in movement patterns between species and ecosystems are to be expected (e.g., see Fig. 3), but for our data these differences did not alter inferences about the importance of step bias (e.g., see Fig. 5A). For Selous, the data came from 1540 observations from 10 packs over a period of 5 years, with GPS locations recorded at intervals averaging 1.13 ± 0.04 (SEM) hours while following dogs during hunting periods at dawn, dusk, and night. For Liuwa, the data came from 1636 observations from one wild dog carrying a GPS collar that recorded fixes at 5.0 ± 0.01 (SEM) hour intervals for 360 days. In both cases, we excluded data from daily periods of inactivity, when predation risk drops close to zero (Creel and Creel 2002). From these data, we determined the distance moved between consecutive fixes, the time interval between the fixes, and the speed of linear movement. From the speed of movement, we calculated the distance travelled over intervals of 0–6 h (encompassing the range of intervals that is typical of GPS collars for large carnivores) and the total area within 1 km of that path. Finally, we estimated step bias by dividing the area of encounter just described by the area within two circles with a radius of 1 km (i.e., the area of encounter identified only by the GPS points at each end of the step). In the Selous, we also recorded odometer readings at each GPS fix while following wild dogs, which allowed us to estimate the ratio of the actual path length to the linear distance between fixes.

In the schematic diagram of Figure 1, step bias is represented by a comparison of the areas in red and green to the area in red only.

### Scatter bias

**Figure 2:**
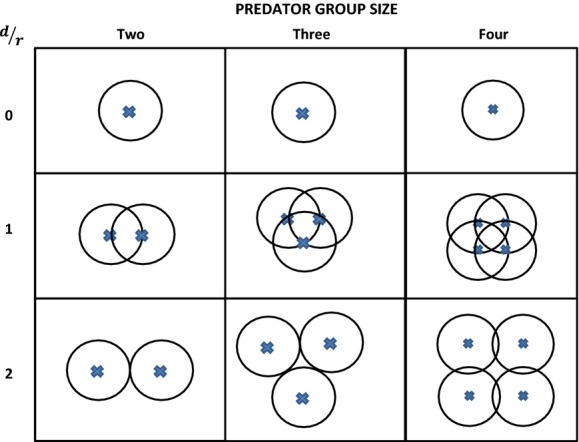
Overlap in the area of prey encounter for predators in groups of two, three, and four, with variation in the mean distance between group mates (*d*) relative to the radius over which they affect prey (*r*).

The area over which a group of predators affects prey is a function of group size (*n*), interindividual distance (*d*), and the “encounter radius” (*r* – the radius over which a predator affects prey behavior). For group sizes up to four predators, this area can be determined by analytic geometry. For *d*/*r* ratios ranging from zero to two, we determined the total area of prey encounter for groups of 2, 3, or 4 predators, using formulae for the area of overlap between two circles with defined radius and separation. For each group size, Figure 2 illustrates *d*/*r* ratios of zero (no scatter among group mates), one (interindividual distance equal to the encounter radius), and two (mean interindividual distance equal to twice the encounter radius). We calculated scatter bias as the ratio of total area for a given combination of *n*, *d,* and *r*, relative to the area of a circle of radius *r*.

In the schematic diagram of Figure 1, scatter bias is represented by a comparison of the areas in red and blue to the area in red only. To put these calculations of scatter bias in context, we used the distribution of interindividual distances between pairs of GPS-collared wolves living in the same pack. For pairs of wolves in three packs, Middleton et al. (2013a) reported that 75% of paired locations were within 0.26, 0.47, and 0.89 km, respectively. Here, we used these values to simulate scatter among individuals by drawing 10,000 pseudorandom replicates from three lognormal distributions with 75th percentiles matching the above values and to obtain bootstrap estimates of *d*/*r* ratios with an encounter radius of one kilometer.

### Total bias in estimated frequency and strength of antipredator responses

For these data, we placed bounds on the total underestimation of predator–prey encounter rates using a four-step process:

- We calculated step bias from the speed of straight-line movements of African wild dogs, for intervals of 0–6 h between GPS locations.
- We calculated the amplification of step bias due to nonlinearity in movement, using the ratio of true path length to straight-line distance between GPS fixes for wild dogs.
- We calculated scatter bias analytically for *d*/*r* ratios ranging from 0 to 2 and group sizes of 1–4.
- We determined total underestimation bias as the product of these three components. In the schematic diagram of Figure 1, total bias is represented by the ratio of areas in red, blue, and green to the area in red only. This schematic does not include the effect of nonlinear movements between GPS fixes.

Underestimation bias in the frequency of encounter should be equal to bias in the estimated area within a defined encounter distance. Underestimation bias in the strength of antipredator responses should be proportional to bias in the estimated area within a defined encounter distance. Underestimation of the frequency of predator–prey encounters is compounded by underestimation of the strength of responses.

## Results

### Step bias

**Figure 3:**
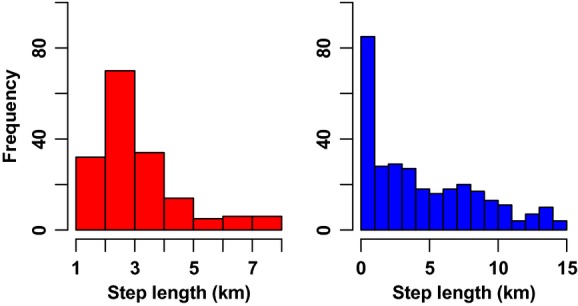
The frequency distribution of straight-line distance moved (step length) over a period of 3 h for African wild dogs in Liuwa National Park (left) and the Selous Game Reserve (right).

The mean speed of linear movement during periods of activity was 1.38 km/h (±0.67 SD) for African wild dogs in Selous and 1.03 km/h (±0.51 SD) in Liuwa. In Figure 3, we show the frequency distribution of linear step lengths for these two populations over an interval of 3 h. In these two populations, mean linear step length for a period of 3 h increased the area within 1 km of the predators by a factor of 2.7x–3.3x, relative to the area within 1 km of the GPS locations themselves (Fig. 5). For wild dogs in Selous, the path actually travelled by wild dogs was longer than the linear distance between GPS locations by a factor of 1.59 ± 0.11 (SEM), increasing the total effect of step bias to 5.3-fold.

### Scatter bias

Figure 2 shows changes in the total area over which a group of predators affects prey, as group size increases and as the mean distance between group mates (*d*) increases, relative to the radius over which predators affect prey (*r*). A *d*/*r* ratio of zero describes a highly cohesive predator group in which all members always remain together. A *d*/*r* ratio of one describes a situation in which the mean distance between group mates is equal to the distance over which they affect prey, so that they have heavily overlapping areas of influence on prey behavior. For groups of three or fewer predators, a *d*/*r* ratio of two describes a situation in which group mates are sufficiently scattered that they have nonoverlapping areas of influence (e.g., white-tailed mongooses or striped hyenas: Waser and Waser 1985; Wagner et al. 2008). It is perhaps counterintuitive that *d*/*r* = 2 is not equivalent to “no overlap in the area of influence among group mates” for predator groups with four or more members. In Figure 2, this lack of equivalence is apparent for the bottom right panel (*d*/*r* = 2 and *n* = 4), where two of the interindividual distances are diagonals that are longer than the other four interindividual distances. For mean *d*/*r* to equal 2, the four shorter interindividual distances must be <2, and thus, the areas of influence must overlap.

**Figure 4:**
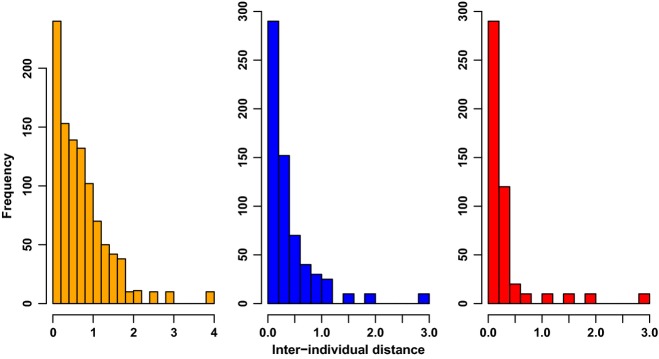
Frequency distributions of interindividual distances drawn from lognormal distributions with 75th percentiles of 0.89 km (left), 0.47 km (middle), and 0.26 km (right), as reported for GPS collared wolves in three packs in the Greater Yellowstone Ecosystem.

**Figure 5:**
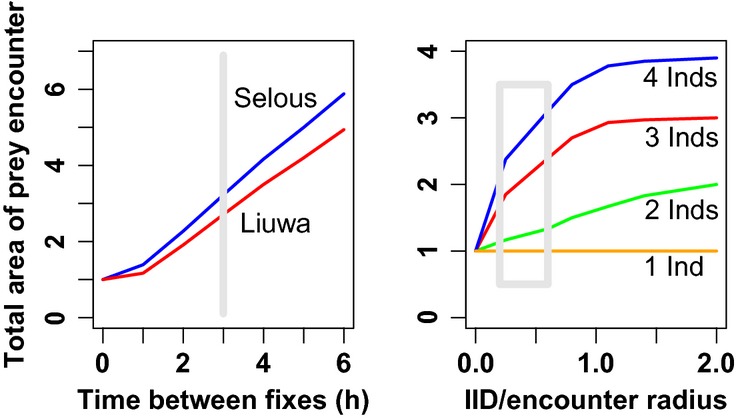
(Left) Step bias, measured as the total area within 1 km of a straight line between two GPS locations, relative to the area within 1 km of the GPS locations themselves. The curves show the magnitude of step bias for mean rate of movement during hunting periods for African wild dogs in two populations (Selous and Liuwa: see Fig. 3), for GPS-collar data with a typical range of fix rates. The gray line identifies the fix rate from Middleton et al. (2013a). (Right) Scatter bias, measured as the increase in total area within which prey is encountered as interindividual distance (*d*) increases, relative to the radius (*r*) over which a predator affects prey. As the number of predators in a group increases, scatter bias becomes strong, even for moderate *d*/*r* ratios. The gray box identifies mean *d*/*r* ratios for interindividual distances from the three wolf populations in Figure 4, relative to an encounter radius of 1 km.

Figure 4 shows distributions of interindividual distance drawn from lognormal distributions matching the 75th percentiles for three wolf packs reported by Middleton et al. (2013a). These distributions can be related directly to Figure 2, because they are equivalent to a frequency distribution of *d*/*r* ratios with an encounter radius of 1 km. Figure 5 (right) shows the increase in scatter bias as the *d*/*r* ratio increases and as group size increases, with gray lines enclosing the observed range of mean *d*/*r* ratios observed for wolves (using means from the distributions in Figure 4). For these distributions of interindividual distance, ignoring scatter among group mates would cause up to threefold underestimation of the area over which predators affect prey, even for group sizes of four or less. The effect of scatter bias increases as predator group size increases, but cannot be calculated analytically for groups larger than four. Because the general inference is clear from Figure 5, we did not use simulation modeling to extend the analysis to larger groups.

### Combined effects on underestimation of the frequency and strength of antipredator responses

Accounting for predator–prey encounters that occur during linear movements between GPS fixes at 3-h intervals increased the estimated rate of interaction between cursorial canids and their prey by a factor of 2.7–3.3. Accounting for the observed nonlinearity of movements increased the estimated encounter rate by a factor of 1.6. Accounting for scatter among individuals increased the estimated encounter rate by a factor of 3, even for relatively small groups of four or fewer. For larger groups, this effect would be stronger. The product of these three biases suggests that cursorial predators induce antipredator responses over an area more than 10 times larger than the area identified by typical data from GPS collars. This calculation is based on an encounter radius of 1 km, but the results are similar for other response thresholds. Unless GPS fixes are collected very frequently and a large proportion of the predator population is collared, uncorrected encounter rates from GPS telemetry will substantially underestimate the nonconsumptive effects of predators on prey (Fig. 5).

## Discussion

Data from African wild dogs and wolves show that step bias and scatter bias can cause large underestimation of the area over which prey is affected by the immediate presence of predators. For studies using locations from GPS collars as the sole source of data on the area affected by predation risk, these biases should be carefully considered. The biases can be reduced in several ways. First, one can reduce scatter bias by increasing the fraction of the population that is collared, giving careful consideration to the distribution of collars within and among groups for group-hunting predators. For species with fission–fusion group dynamics, a large proportion of the population must be collared to avoid scatter bias. Second, one can reduce step bias by increasing the rate at which GPS collars record locations. Third, one can use direct observation of radiocollared predators to add to the information about predation risk, for instance by confirming which group members are present, whether they are actively hunting, the presence of recent kills, etc. (e.g., Liley and Creel 2008). Finally, one can aggregate GPS locations over a period of time to produce a “landscape” of predation risk (Laundré et al. 2001; Thaker et al. 2011): This approach still requires careful consideration of the possibility that apparent variation in risk across the landscape is driven by variation in sampling intensity due to the distribution of collars within the population.

### Effects of predation risk on elk

To illustrate the importance of methodological underestimation bias for inferences about the strength of risk effects, we examine a recent study that relied solely on encounter data from GPS collars to measure the effects of predation risk on elk. Using data from two adjacent Wyoming elk herds that differed in exposure to predation risk, Middleton et al. (2013a) found that “when wolves approached within 1 km, elk increased their rates of movement, displacement, and vigilance”, but inferred that the frequency and strength of responses to wolves were too small to cause important effects on elk demography or dynamics. Middleton et al. estimated the frequency and strength of antipredator responses using wolf locations from GPS collars on ∼30% of the population, recording locations at 3-h intervals. Our results (Fig. 5) show that such methods are expected to underestimate the frequency and strength of antipredator responses by a factor of ∼10.

**Figure 6:**
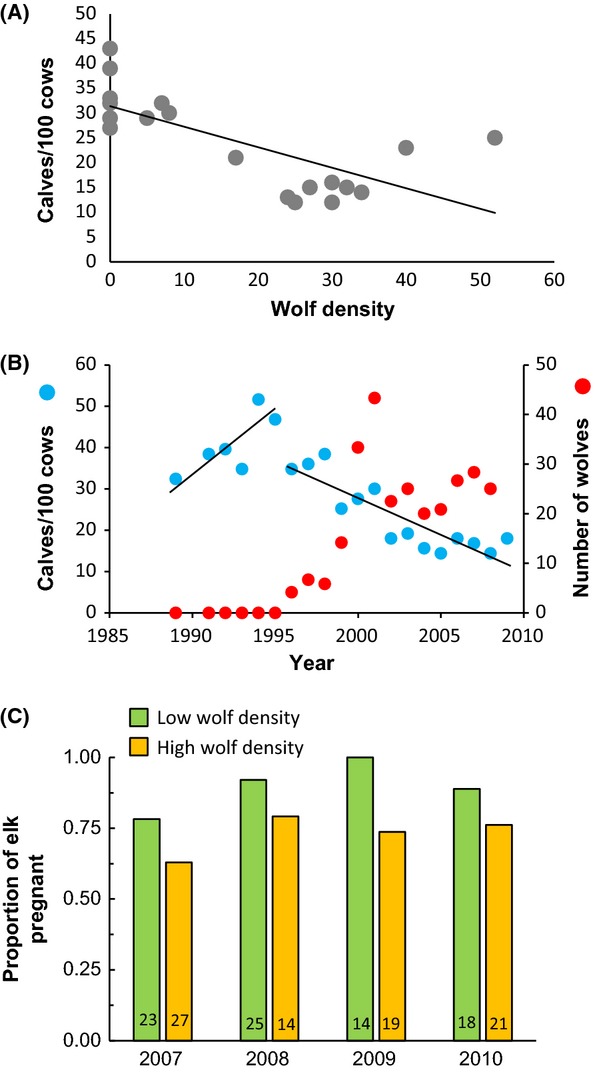
(A) Calf recruitment in the elk population exposed to high predation risk in Middleton et al.'s study was negatively related to wolf density, as has been found in most other GYE elk populations. Wolf density explained more than half of the observed variation in elk calf recruitment. (B) In the migratory elk herd colonized by wolves in Middleton et al.'s study, winter calf recruitment began declining abruptly at the time of wolf recolonization. A breakpoint regression model with a change of sign at the year of wolf recolonization provided a significantly better fit than a linear model of continuous decline due to climate change, as has been suggested by Middleton et al. (2013a) (adjusted *r*^2^ = 0.89 (breakpoint model); adjusted *r*^2^ = 0.67 (linear model); nested ANOVA, *F* = 5.98, *P* = 0.011, ΔAICc > 4). It would be unprecedented for climate change to abruptly cause a large decline in reproduction within this herd with no detectable effect on a spatially overlapping herd (Middleton et al. 2013b) or other herds in the region, for which climate conditions over this interval yielded continued growth (Creel and Creel 2009). (C) Elk pregnancy rates were significantly lower in the herd exposed to high wolf density, when compared with the immediately adjacent herd with little exposure to wolves (*z* = 2.72, *P* = 0.0066 by GLM; ΔAICc > 2 for models including effects of year and interaction). Numbers within bars are sample sizes. Data from Wyoming Department of Fish and Game (wgfd.wyo.gov) and USGS Wyoming Co-op Unit (http://www.wyocoopunit.org).

**Figure 7:**
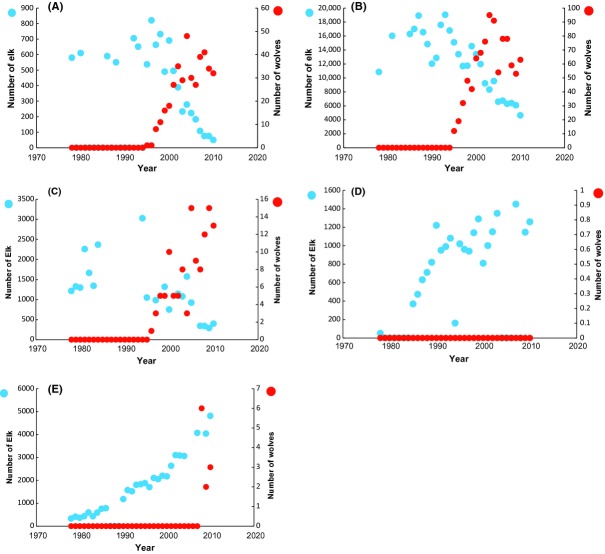
Elk population dynamics in relation to recolonization by wolves for five major segments of the Greater Yellowstone Ecosystem: (A) Madison–Firehole, (B) Northern Range, (C) Upper Gallatin. In these populations, elk numbers began declining immediately after wolf recolonization, while elk populations generally remained stable or continued to grow in adjacent locations that were not recolonized or maintained a small and sporadic local wolf population, as illustrated by (D) the Tobacco Root Range and (E) the Crazy Mountains. Original data from annual reports by U.S. Fish and Wildlife Service, Montana Department of Fish, Wildlife and Parks, U.S. National Park Service and citations in text.

Bias of this magnitude in estimates of exposure to predation risk would be expected to affect inferences about the demographic costs of antipredator responses. For example, the analyses summarized in Fig. 5 show that the conclusion that “encounters occurred only once every 9 days” (Middleton et al. 2013a) is based on sampling that is likely to detect only a small fraction of encounters. The relationship between wolf encounter rates and pregnancy was further obscured by missing data: for 29% of elk whose pregnancy status was determined, gaps in wolf GPS coverage meant that no data were available for the rate of encounter with wolves. In these cases, Middleton et al. (2013a) replaced the missing data with “encounter days observed during other winters”, effectively testing the hypothesis that predation risk in past or future years affects current pregnancy rates. Straightforward data on the pregnancy rates of the two herds studied by Middleton et al. (2013a) show that pregnancy rates were 24% lower in the herd exposed to higher predation risk (68% vs. 89%: Stephenson 2010; or 70.6% vs. 89.2%: Middleton et al. 2013a; *z* = 2.72, *P* = 0.007). For the herd exposed to higher wolf predation risk, pregnancy rates and calf recruitment decreased abruptly at the time of wolf recolonization (Fig. 6), as predicted a priori by the hypothesis that predation risk affects elk reproduction (Creel et al. 2007). Potential changes in the age structure of the herd do not provide an explanation for the observed decrease in pregnancy rates, because wolves prey preferentially on calves and senescent females with low reproductive potential (Wright et al. 2006), which should cause an increase in per capita birth rates rather than the observed decrease. Indeed, Middleton et al. (2013a) reported that exposure to predation risk was associated with lower pregnancy rates after controlling for age, and that pregnancy rates were lower in the herd exposed to higher predation risk for every age-class (see Fig. 3A in Middleton et al. 2013b). Including the population studied by Middleton et al. (2013a), pregnancy rates have now been found to decrease (by 24–43%) with increased predation risk in 10 elk populations widely distributed across western North America. (Zager et al. 2005; Creel et al. 2007, 2011; Garrott et al. 2009; Stephenson 2010). These observed decreases in pregnancy rate have been substantially larger than the additive mortality due to wolf predation (Creel et al. 2011; Griffin et al. 2011; Brodie et al. 2013), suggesting that risk effects contribute importantly to observed population declines (e.g., see Fig. 7 below). To our knowledge, the hypothesis that elk pregnancy rates decline when predation risk increases has been tested and supported more broadly than any other demographic risk effect among vertebrates.

When individuals of a long-lived, highly iteroparous species encounter an energetic constraint such as increased predation risk, natural selection favors reduced reproductive effort to avoid effects on body condition and adult survival (Gaillard et al. 1998; Fisher 1999; Eberhardt et al. 2003), just as Middleton et al. (2013a) observed. Progesterone levels of elk are negatively correlated with the risk of predation (Creel et al. 2007, 2011), and Middleton et al. (2013a) data confirm that by reducing reproductive effort, elk can avoid loss of fat stores and increased mortality (as in other ungulates: Gaillard et al. 1998). Increased vigilance and reduced foraging are ubiquitous antipredator responses (Brown and Kotler 2004) and have been observed in several elk populations (Childress and Lung 2003; Winnie and Creel 2007), with effects on diet and nutrition (Christianson and Creel 2008, 2010). That such responses yield a decrease in reproduction (Fig. 6) rather than fat stores do not logically support an inference that the costs of antipredator responses are small. A decrease in energy intake (Christianson and Creel 2010) is not directly synonymous with a decrease in fat stores; if elk exposed to high predation risk in Middleton et al. 's (2013a)study had maintained body fat without a large reduction in reproductive rate, the data would support their inference that risk effects are weak. However, 32% of adult female elk in the herd exposed to wolf predation were not pregnant (Stephenson 2010) and thus could redirect the saved energy into maintenance of body fat. Only 11% of the adult females in the adjacent herd exposed to low predation risk were not pregnant. To ignore this substantial difference in pregnancy rates when examining data on body fat is to implicitly assume that gestation carries no energetic cost, but Pekins et al. (1998) used resting calorimetry to show that the energy demand of cervids increases by 45% during the last trimester of pregnancy. In short, a comparison of adult female body fat late in gestation that ignores large differences in pregnancy rates (as in Middleton et al. 2013a) will inevitably yield misleading results. Middleton et al. (2013a) also compared mean body fat to populations in other regions, but did not control for large differences in the date of sampling or winter conditions, which have effects strong enough to render this comparison very weak as a test of risk effects (Creel et al. 2011).

Middleton et al. (2013a) argued that “the ecological consequences of actively hunting large carnivores, such as the wolf, are more likely transmitted by consumptive effects on prey survival” than by risk effects. This inference is contradicted by extensive data showing that the additive effects of direct wolf predation are too small to account for observed changes in elk dynamics following wolf recolonization. For 2746 radiocollared elk in 45 populations, Brodie et al. (2013) found that “wolves and all carnivore species combined had additive effects on baseline elk mortality, but only reduced survival by <2%”. For 1999 radiocollared calves in 12 populations, Griffin et al. (2011) found that “wolf predation was low and most likely a compensatory source of mortality”. When direct predation has little effect on survival rates, as in these extensive data for elk, risk effects are expected to comprise a large fraction of predation's limiting effect (Creel 2011).

Collectively, these studies reveal a coherent pattern across many elk populations: (1) Numbers decreased abruptly at the time of wolf recolonization, while most noncolonized populations (and populations where wolf numbers were held down by predator control operations) experienced continued growth (Fig. 7; Hebblewhite et al. 2002; Creel and Creel 2009; Garrott et al. 2009). (2) Population declines were larger than can be explained by the additive effects of direct predation (Griffin et al. 2011; Brodie et al. 2013). (3) Population declines aligned closely with abrupt decreases in pregnancy rates and calf recruitment (Creel et al. 2007, 2011; Garrott et al. 2009).

Increased predation by grizzly bears might contribute to reduced recruitment in some elk populations, but bears hibernate for most of gestation and pose little threat to adult elk, so they are a poor explanation for reduced pregnancy rates. Climate change may affect elk reproduction, but Garrott et al. (2009) found “little support for either a cold- or warm-season climate effect” on calf recruitment in postwolf data from Yellowstone. At the regional scale, weak costs of recently decreased summer precipitation on elk population dynamics were more than offset by strong benefits of decreased snow accumulation (Creel and Creel 2009). Considering the spatial and temporal details of changes in pregnancy rates and recruitment within and among populations (e.g., Figs. 6 and 7), risk effects provide a more coherent explanation than these alternative hypotheses (Creel et al. 2007, 2011).

Current theory does not establish that “the wolf is not predicted to induce antipredator behaviurs strong enough to impact prey demography” as argued by Middleton et al. (2013a). Middleton et al. (2013a) assumed that cursorial predators induce weaker antipredator responses because their locations are less predictable than those of ambush predators, but this assumption remains largely untested for vertebrates (but see Thaker et al. 2011). One can make a logical counterargument that selection against predictability should be stronger for ambush predators, whose hunting success depends on surprise. Cursorial hunters such as wolves and wild dogs do not rely on surprise when initiating a hunt and are normally more successful when prey flees than when they do not (Creel and Creel 2002). Active predators might cause weaker demographic risk effects than sit-and-wait predators (Preisser et al. 2007; Schmitz 2008), but this has not been empirically tested for any vertebrate. Cursorial hunters have exceptionally large energetic costs of hunting (Gorman et al. 1998), and it is reasonable to hypothesize that the costs of avoiding such predators are also large. A broad range of behavioral, ecological, physiological, and demographic data (now from 10 populations) suggests that predation risk reduces reproduction, with strong effects on population dynamics of elk. Given the strength and ubiquity of inducible defenses such as vigilance, such risk effects are probably common.
